# A Review and Meta-Analysis of the Safety and Efficacy of Using Glucagon-like Peptide-1 Receptor Agonists

**DOI:** 10.3390/medicina60030357

**Published:** 2024-02-21

**Authors:** En-Hao Hu, Ming-Lung Tsai, Yuan Lin, Tien-Shin Chou, Tien-Hsing Chen

**Affiliations:** 1 Department of Internal Medicine, Keelung Chang Gung Memorial Hospital, Keelung 204, Taiwan; howard8288@cgmh.org.tw; 2 Division of Cardiology, Department of Internal Medicine, New Taipei Municipal TuCheng Hospital, Taipei 236, Taiwan; 3 College of Medicine, Chang Gung University, Taoyuan 333, Taiwan; 4 Department of Emergency Medicine, Keelung Chang Gung Memorial Hospital, Keelung 204, Taiwan; 5 Division of Gastroenterology, Department of Internal Medicine, Keelung Chang Gung Memorial Hospital, Keelung 204, Taiwan; 6 Division of Cardiology, Department of Internal Medicine and the Center of Data Science and Biostatistics, Keelung Chang Gung Memorial Hospital, Keelung 204, Taiwan

**Keywords:** GLP-1, diabetes, insulin, cardiovascular, renal

## Abstract

Glucagon-like peptide-1 receptor agonists (GLP-1 RAs) have been used to reduce glucose levels in patients with type 2 diabetes mellitus since 2005. This meta-analysis discusses the mechanisms and potential benefits of several GLP-1 RAs. In particular, this meta-analysis focuses on the safety and associations with weight loss, glucose reduction, cardiovascular outcomes, heart failure, and renal outcomes of GLP-1 RAs to determine their benefits for patients with different conditions. In terms of glycemic control and weight loss, semaglutide was statistically superior to other GLP-1 RAs. In terms of cardiovascular outcomes, 14 mg of semaglutide taken orally once daily and 1.8 mg of liraglutide injected once daily reduced the incidence of cardiovascular death, whereas other GLP-1 RAs did not provide similar benefits. Moreover, semaglutide was associated with superior outcomes for heart failure and cardiovascular death in non-diabetic obesity patients, whereas liraglutide worsened heart failure outcomes in diabetic patients with a reduced ejection fraction. Additionally, semaglutide, dulaglutide, and liraglutide were beneficial in terms of composite renal outcomes: These GLP-1 RAs were significantly associated with less new or persistent macroalbuminuria, but not with improved eGFR deterioration or reduced requirement for renal replacement therapy. However, GLP-1 RAs may benefit patients with type 2 diabetes mellitus or obesity.

## 1. Introduction

Type 2 diabetes mellitus (T2DM) has been steadily increasing in prevalence globally. In response to this critical health concern, researchers have developed novel oral glucose-lowering agents, including glucagon-like peptide-1 receptor agonists (GLP-1 RAs) [1,2]. GLP-1 RAs can reduce glycated hemoglobin (Hb1Ac), improving glycemic control and reducing weight [3]. Additionally, GLP-1 RAs are associated with a low risk of hypoglycemic episodes [4]. Moreover, according to the 2023 guidelines of the American Diabetes Association, GLP-1 RAs are associated with numerous cardiovascular benefits in patients with T2DM with comorbidities of established atherosclerotic cardiovascular diseases (ASCVDs) [5]. This review introduces the mechanisms, development history, and current clinical applications of GLP-1 RAs [6].

## 2. Physiology of Glucagon-like Peptide-1 (GLP-1)

GLP-1 is produced in enteroendocrine L-cells of the distal small bowel and colon. Concentrations of GLP-1 are low during fasting and high after meals [7]. GLP-1 is produced in the intestines after food intake and activated by DPP-4 enzyme cleavage. Although GLP-1 is released rapidly, it has a short half-life of approximately 2 min [8]. After DPP-4 cleavage, GLP-1 is released biphasically. During the rapid first phase, GLP is released into the bloodstream within 15–30 min of nutrient ingestion. In the second, more gradual phase, a minor peak in bloodstream GLP-1 levels occurs between 90 and 120 min after nutrient ingestion. Multiple agents have been demonstrated to affect GLP-1 secretion, consisting of fat, gamma-aminobutyric acid, glycine, and somatostatin [9,10].

As GLP-1 binds to its receptors, adenylate cyclase is activated, elevating cyclic adenosine monophosphate (cAMP) levels. Elevated cAMP levels increase the concentrations of protein kinase A (PKA) and cAMP-regulated guanine nucleotide exchange factor 2 (cAMP-GEF2), causing a cascade of reactions that ultimately increases cytoplasmic Ca^+2^, inducing exocytotic insulin release from insulin granules and mitochondrial adenosine triphosphate (ATP) synthesis [11,12].

In normal beta cells, insulin is influenced by blood sugar levels in a linear fashion. Insulin release occurs in two phases. During the first phase, which lasts approximately 10 min, hepatic glucose production is suppressed. During the second phase, which lasts approximately 2 h, insulin is released into the bloodstream. However, in patients with T2DM, the first phase does not occur or occurs with hepatic glucose suppression, delaying and impeding the onset of the second phase and allowing increased insulin levels to accumulate in the bloodstream. Excess insulin production to overcome insulin resistance causes healthy beta cells to be gradually replaced with amyloids [13,14]; once patients are clinically diagnosed with T2DM, they retain only approximately 50% of normal beta cell function [15]. A decline in beta cell function contributes to the failure of many biological processes that promote long-term glycemic control [16]. GLP-1 RAs counteract this decline by improving insulin resistance and glucose homeostasis [17].

GLP-1 is also associated with weight loss in clinical trials [18,19,20,21]. The mechanism through which GLP-1 induces weight loss involves several hypothalamic nuclei (the arcuate nucleus of the hypothalamus, the periventricular hypothalamus, and the lateral hypothalamic area) and hindbrain nuclei (the parabrachial nucleus and the medial nucleus tractus solitarius), in addition to the hippocampus (the ventral subregion) and the nuclei embedded within the mesolimbic reward circuitry (the ventral tegmental area and the nucleus accumbens) [22]. One study conducted two experiments demonstrating that the activation of GLP-1 in the subdiaphragmatic vagal afferents and the brain contributes to the intake-inhibitory effects of GLP-1 RAs [23]. Consequently, GLP-1 RAs suppress metabolism and appetite, leading to body weight loss.

## 3. General Effects and Developments

GLP-1 regulates blood sugar levels by promoting insulin production and inhibiting glucagon. Additionally, GLP-1 limits weight gain by suppressing gastric emptying and appetite [24,25]. The first GLP-RA to be available commercially was exenatide, approved in 2005 by the United States Food and Drug Administration (US FDA) [26]. The second GLP-1 RA to be commercially available was liraglutide, approved in 2009, which was designed to be similar to mammalian GLP-1 [27]. Liraglutide binds free fatty acids to plasma albumin and intestinal fluids; the resulting albumin-bound reservoir prolongs the medication’s effects. Moreover, liraglutide has an elimination half-life of approximately 13 h, making it suitable for once-daily injection [28,29]. Subsequent advances in GLP-1 RAs extended this half-life, utilizing large protein-bound compounds such as dulaglutide or efpeglenatide (bound to immunoglobulin Fc fragments), and albiglutide (bound to albumin) [30,31,32]. These GLP-1 compounds degrade slowly, with half-lives of approximately 1 week, enabling a once-weekly injection [28]. Another GLP-1 RA, semaglutide, has a modified chemical structure that promotes binding to albumin, enabling a similar week-long half-life. Additionally, semaglutide was the first GLP-1 RA approved for oral administration [33].

Table 1 presents a summary of the characteristics of these GLP-1 RAs, including pharmacokinetics, dosing frequency, and administration.

## 4. Reduction in Blood Glucose Levels and Weight

We identified eight trials evaluating the efficacy of GLP-1 RAs in reducing glucose levels and weight in patients with T2DM: DURATION-1, LEAD-6, DURATION-5, DURATION-6, HARMONY-7, AWARD-6, SUSTAIN-3, and SUSTAIN-10. The levels of HbA1c and weight reduction observed in patients taking the GLP-1 RA regimens in these trials are presented in Table 2.

DURATION-1 was a 30-week randomized control trial that demonstrated that 2 mg of exenatide once weekly yielded superior outcomes in reducing HbA1c (−1.9% vs. −1.5%, *p* = 0.0023) but similar outcomes in weight reduction (−3.7 kg vs. −3.6 kg, *p* = 0.89) relative to 10 μg of exenatide twice daily [43]. These results invite comparison with DURATION-6, a 26-week, open-label, randomized, parallel-group study. In this trial, liraglutide (administered once weekly) was compared with exenatide (administered twice daily), with the primary endpoint being HbA1c change. Liraglutide was associated with a significantly greater reduction in HbA1c levels than exenatide (−1.48% vs. −1.28%, *p* = 0.02). In terms of body weight decrease, the liraglutide group was also superior to the exenatide once-weekly group (−3.57 kg vs. −2.68 kg, *p* = 0.0005) [39]. A separate trial, DURATION-5, was an open-label, randomized study comparing exenatide injected once weekly with exenatide injected twice daily over a period of 24 weeks. The main outcome measure was the changes in HbA1c levels. Exenatide administered once weekly was associated with significantly greater reductions in HbA1c levels than exenatide administered twice daily (−1.6% vs. −0.9%, *p* < 0.0001). In terms of weight loss, exenatide once weekly was associated with greater weight loss than exenatide twice daily (−2.3 kg vs. −1.4 kg, *p* < 0.05) [37]. Based on these trials, this study’s analysis concluded that liraglutide administered once weekly was associated with significantly lower HbA1c levels and weight than 2 mg of exenatide once weekly and 10 ug of exenatide twice daily.

The LEAD-6 trial, a 26-week open-label, parallel-group, multinational study, compared the administration of 10 ug exenatide twice daily with 1.8 mg liraglutide once weekly. The primary outcome of the study was a change in HbA1c levels. Liraglutide reduced HbA1c significantly more than exenatide twice daily (−1.12% vs. −0.79%, *p* < 0.0001). Liraglutide was also associated with significantly more weight loss than exenatide 10 ug twice daily (−3.24 kg vs. −2.87 kg, *p* = 0.0005) [38]. Liraglutide was also studied in HARMONY-7, a 32-week, open-label, phase 3, noninferiority study comparing albiglutide once weekly with liraglutide once weekly. The primary endpoint was a change in HbA1c levels. The study found no significant difference between liraglutide and albiglutide once weekly (−0.99% vs. −0.78%, *p* = 0.0846) for this outcome. However, liraglutide was associated with more weight loss than albiglutide (−2.16 kg vs. −0.64 kg, *p* < 0.0001) [40]. Liraglutide also performed well in another trial, AWARD-6. In this phase 3, randomized, open-label, parallel-group study comparing 1.5 mg of dulaglutide with 1.8 mg of liraglutide with a primary outcome of noninferiority of dulaglutide with liraglutide with respect to change in HbA1c levels, dulaglutide and liraglutide were associated with reductions in HbA1c of −1.42% and −1.36%, respectively (noninferiority *p* value < 0.0001), which met the noninferiority criteria defined by the study. Liraglutide was associated with significantly greater weight loss than dulaglutide (−3.61 kg vs. −2.90 kg, *p* = 0.011) [41]. In summary, with respect to lowering HbA1c levels, liraglutide was just as effective as albiglutide, dulaglutide, and exenatide twice daily. Moreover, liraglutide was associated with significantly more weight loss than dulaglutide, albiglutide, and twice daily exenatide.

The administration of 1 mg of semaglutide once weekly was compared with the administration of 2 mg of exenatide once weekly in SUSTAIN-3, a 56-week, phase 3a, open-label, parallel-group, randomized controlled trial. The primary endpoint was a change in HbA1c levels. The results revealed that semaglutide reduced HbA1c significantly more than exenatide (−1.5% vs. −0.9%, *p* < 0.0001). Additionally, the semaglutide group experienced greater weight loss than the exenatide group (−5.6 kg vs. −1.9 kg, *p* < 0.0001) [20]. Semaglutide was further evaluated in SUSTAIN-10, a 30-week, phase 3b, open-label trial comparing 1 mg of semaglutide once weekly with 1.8 mg of liraglutide daily. The primary outcome was a change in HbA1c levels from baseline. Semaglutide was associated with significantly greater reductions in HbA1c levels than liraglutide (−1.7% vs. −1.0%, *p* < 0.0001). Moreover, semaglutide was associated with significantly greater weight loss than liraglutide (−5.8 kg vs. −1.9 kg, *p* < 0.0001) [42]. In summary, 1 mg of semaglutide administered weekly was more effective than 2 mg of exenatide administered once weekly and 1.8 mg of liraglutide administered once daily.

After comprehensively reviewing the data on these GLP-1 RAs, we observed that in terms of glycemic control, the GLP-1 RAs could be ranked in descending order of effectiveness as follows: 1 mg of semaglutide, 1.8 mg of liraglutide, 1.5 mg of dulaglutide, 50 mg of albiglutide, 2 mg weekly of exenatide, and 10 µg twice daily of exenatide. The same relative order of superiority was observed in terms of associated weight loss. However, more and larger comparison trials are required to determine the efficacy of these various GLP-1 RAs.

## 5. Cardiovascular Effects

GLP-1 RAs yield improvements in cardiovascular (CV) outcomes due to their insulinotropic blood pressure reduction and weight-lowering action [44,45,46]. Moreover, in rodents, GLP-1 RAs increased cardiomyocyte survival by inhibiting apoptosis, improving regional and global cardiac output following injury or heart failure, and ameliorating endothelial dysfunction [47,48,49].

According to the American Diabetes Association guidelines from 2023, GLP-1 RAs offer several CV benefits to patients with T2DM and ASCVDs. According to them, Dulaglutide, liraglutide, and semaglutide can prevent major adverse cardiovascular events (MACEs), whereas exenatide and lixisenatide had no effect on the occurrence of MACEs [5]. Accordingly, the European Society of Cardiology guidelines from 2023 recommend sodium-glucose cotransporter 2 inhibitors (SGLT-2is) and GLP-1 Ras as a preferred glucose-lowering therapy for patients with T2DM and ASCVD [50].

Table 3 presents the basic characteristics of the trials and their primary composite cardiovascular (CV) outcomes; these trials compared GLP-1 RAs against placebos. With regard to three-point MACEs (CV-related death, myocardial infarction, and stroke), liraglutide, semaglutide, albiglutide, and dulaglutide significantly reduced the risks of all three MACES. Exenatide and semaglutide (oral) reduced MACE risk but not significantly so. Moreover, in the ELIXA study, lixisenatide did not reduce MACE risk. Compared with placebos, liraglutide and oral semaglutide significantly reduced the risk of CV-related death. By contrast, injection of dulaglutide, albiglutide, lixisenatide, exenatide, and semaglutide did not significantly reduce the risk of CV-related death. Myocardial infarction risk was significantly reduced by liraglutide and albiglutide; non-significantly reduced by dulaglutide, exenatide, lixisenatide, oral semaglutide, and semaglutide injection; and not reduced by lixienatide or oral semaglutide. Stroke risk was significantly reduced by only dulaglutide and non-significantly reduced by all other GLP-1 RAs except lixienatide. Table 3 lists the individual hazard ratios for three-point MACEs in each trial.

In the EXSCEL trial, once-weekly exenatide exhibited significant noninferiority to placebo in three-point MACEs (hazard ratio: 0.91; 95% confidence interval [CI]: 0.83–1.00). However, with respect to the individual components of three-point MACES—cardiovascular-related death, myocardial infarction, and stroke—once-weekly exenatide did not differ significantly compared with placebos [51]. In the LEADER trial, liraglutide had significantly fewer three-point MACEs (hazard ratio: 0.87; 95% CI: 0.78–0.97; *p* < 0.001 for noninferiority; *p* = 0.01 for superiority). Liraglutide was also significantly superior to placebos with respect to CV-related death (hazard ratio: 0.78; 95% CI: 0.66–0.93; *p* = 0.007) and myocardial infarction (hazard ratio: 0.86; 95% CI: 0.73–1.00; *p* = 0.046). With respect to stroke, liraglutide was similar to placebos [52]. In the SUSTAIN-6 trial, subcutaneous semaglutide was associated with significantly fewer three-point MACEs (hazard ratio: 0.74; 95% CI: 0.58–0.95; *p* < 0.001 for noninferiority; *p* = 0.02 for superiority). However, the differences with respect to individual MACES were not significant compared with placebos [53]. Additionally, in the ELIXA trial, the lixisenatide group had significant noninferiority to the placebo group with respect to three-point MACEs (*p* < 0.001) but did not exhibit superiority (*p* = 0.81) (hazard ratio: 1.02; 95% CI: 0.89–1.17). No significant difference was observed between the two groups with respect to the individual MACEs [54]. Furthermore, in the Harmony Outcomes trial, albiglutide exhibited significant superiority to placebo with respect to three-point MACEs (hazard ratio: 0.78, 95% CI: 0.68–0.90) (*p* < 0.0001 for noninferiority; *p* = 0.0006 for superiority). Albiglutide was also associated with significantly improved outcomes with respect to myocardial infarction (hazard ratio: 0.75; 95% CI: 0.61–0.90; *p* = 0.003). With respect to stroke and CV-related death, no significant differences were observed [55]. Additionally, in the REWIND trial, dulaglutide was associated with significantly fewer three-point MACEs (hazard ratio: 0.88: 95% CI: 0.79–0.99; *p* = 0.026). Moreover, in terms of stroke, dulaglutide exhibited significant superiority to placebos (HR: 0.76, 95% CI: 0.62–0.94; *p* = 0.010). However, with respect to myocardial infarction and CV-related death, no significant differences were observed [56]. Furthermore, in the PIONEER-6 trial, oral semaglutide exhibited significant noninferiority to placebo with respect to three-point MACEs (hazard ratio: 0.79; 95% CI: 0.57–1.11; *p* < 0.001 for noninferiority). Oral semaglutide was also associated with a significantly lower risk of CV-related deaths (hazard ratio: 0.49; 95% CI: 0.27–0.92); *p* = 0.021). However, with respect to stroke and myocardial infarction, no significant differences were observed [57]. In another trial (AMPLITUDE-O), efpeglenatide was associated with significantly fewer three-point MACEs (hazard ratio: 0.73; 95% CI: 0.58–0.92; *p* < 0.001 for noninferiority; *p* = 0.007 for superiority). The results for other components of the primary outcome pertained to CV-related death (hazard ratio: 0.49; 95% CI: 0.27–0.92, *p* = 0.07), nonfatal myocardial infarction (hazard ratio: 1.18; 95% CI: 0.73–1.90, *p* = 0.09), and nonfatal stroke (hazard ratio: 0.74; 95% CI: 0.35–1.57 *p* = 0.19) [56].

A pooled meta-analysis of the ELIXA, LEADER, SUSTAIN-6, EXSCEL, Harmony Outcomes, REWIND, PIONEER 6, and AMPLITUDE-O trials conducted by Lancet Diabetes Endocrinol 2021 revealed that GLP-1 RAs resulted in a 14% relative risk reduction for three-point MACES compared with placebos (hazard ratio: 0.86, 95% CI: 0.80–0.93; *p* < 0.0001). In individual measures of the three-point MACEs, GLP-1 RAs contributed to a reduction in risk of death from CV causes (hazard ratio: 0.87; 95% CI: 0.80–0.94; *p* = 0.0010), myocardial infarction (hazard ratio: 0.90; 95% CI: 0.83–0.98; *p* = 0.020), and stroke (hazard ratio: 0.83; 95% CI: 0.76–0.92; *p* = 0.0002). Moreover, when ELIXA was excluded from the meta-analysis, overall CV benefits of GLP-1 RAs would modestly increase. These results provide further evidence that GLP-1 RAs reduce the occurrence of three-point MACEs and each of their components.

An analysis of these randomized controlled trials (ELIXA, LEADER, SUSTAIN-6, EXSCEL, Harmony Outcomes, REWIND, PIONEER 6, and AMPLITUDE-O) reveals that liraglutide, dulaglutide, albiglutide, semaglutide, and efpeglenatide were associated with statistically significant reductions in three-point MACEs. With respect to individual MACEs, liraglutide and oral semaglutide were significantly associated with reductions in CV-related deaths, aliraglutide and albiglutide were associated with significant reductions in the incidence of myocardial infarctions, and only liraglutide was associated with a significant reduction in the incidence of stroke. One explanation for these findings is that these typical GLP-1 RAs exhibit the ‘’classic effect’’ for their medication type and thus had similar influences on CV outcomes.

## 6. Heart Failure

With respect to heart failure (HF), many hypoglycemic agents, such as thiazolidinediones, exert a strong influence on the risk of developing HF requiring hospitalization. GLP-1 RAs may also affect left ventricular ejection fraction (LVEF) and HF with preserved ejection fraction (HFpEF). The results of meta-analyses of phase-II/III trials (for exenatide, albiglutide, dulaglutide, and liraglutide) revealed that GLP-1RAs were not associated with increased risk of hospitalization for HF, indicating their safety in patients who also have CVD. Three large prospective cardiovascular outcome trials (ELIXA [on lixisenatide], LEADER [on liraglutide], and SUSTAIN-6 [on semaglutide]) have further demonstrated the low risk of HF associated with GLP-1 RAs [31]. Moreover, the STEP-HFpEF trial indicated that semaglutide improved physical functioning (as assessed by 6-min walk distance), preserved HFpEF, and reduced weight in patients with HF and obesity [58]. However, randomized controlled trials of liraglutide (LIVE and FIGHT) and Exenatide (EXSCEL) indicated that these medications increased the risk of hospitalization in patients with HF with a reduced ejection fraction [59,60,61]. The increased risk of hospitalization observed in these trials may be attributable to differences in these medications’ effects on LVEF. LVEF was observed to be 57%, 33%, and 27% in the STEP-HFpEF, LIVE, and FIGHT trials, respectively. Another trial (the SELECT trial) targeted patients with obesity but without T2DM. This trial indicated that semaglutide was significantly superior to placebos in reducing the occurrence of MACEs (hazard ratio: 0.80; 95% CI: 0.72–0.90; *p* < 0.001) [62].

In conclusion, semaglutide reduced HF and the risk of CV events in non-diabetic patients with obesity. However, liraglutide and exenatide increased hospitalization in diabetic patients with HF and a reduced ejection fraction. This meta-analysis found no evidence indicating the effects of other GLP-1 RAs on HF in patients with T2DM or obesity.

## 7. Renal Effects

GLP-1 RAs may improve kidney function through direct or indirect mechanisms. One study suggested that the signaling pathway activated by the binding of GLP-1 and GLP-1 receptors results in the phosphorylation of PKA consensus sites at the NHE3 COOH-terminal region. Once the PKA consensus sites have been phosphorylated, sodium, bicarbonate, and water reabsorption are decreased through the inhibition of NHE3-mediated Na+/H+ exchange in the proximal tubule [9]. Additionally, studies have suggested that the observed renal benefits of GLP-1 RAs stem from indirect interactions between the nervous system [63] and the renin–angiotensin system (RAS) [64], in addition to the regulation of atrial natriuretic peptides (ANPs) [65].

According to the American Diabetes Association guidelines from 2023, GLP-1 RAs, especially liraglutide, dulaglutide, and semaglutide, were associated with beneficial renal outcomes in CV outcome trials; these renal benefits were driven by new onset or persistent macroalbuminuria outcomes [5].

Evidence for the renal benefits of GLP-1 RAs was available for semaglutide, dulaglutide, and liraglutide. We found no complete data on the subgroups of composite renal outcomes for other GLP-1 RAs, (exenatide, lixisenatide, albiglutide, efpeglenatide, and oral semaglutide). Available data are presented in Table 4. With regard to liraglutide, the LEADER trial indicated that this GLP-1 RA was associated with lower rates of the development and progression of diabetic kidney disease than placebos. These results were based on the secondary renal outcomes of the trial, which indicated fewer participants in the liraglutide group than in the placebo group (268 of 4668 patients vs. 337 of 4672; hazard ratio: 0.78; 95% CI: 0.67–0.92; *p* = 0.003) Liraglutide was also associated with significant decreases in the incidence of macroalbuminuria (161 of 4668 patients vs. 215 of 4672; hazard ratio: 0.74; 95% CI: 0.61 to 0.91; *p* = 0.004). However, with respect to the sustained doubling of serum creatinine and the requirement for continuous renal replacement therapy, liraglutide was not associated with significant reductions in these outcomes [52,66]. With regard to the renal outcomes of dulaglutide in patients with T2DM, an exploratory analysis of the REWIND randomized placebo-controlled trial revealed that fewer negative composite renal outcomes were associated with long-term use of dulaglutide compared with placebo (hazard ratio: 0.85; 95% CI: 0.77–0.93; *p* = 0.0004). Moreover, dulaglutide was associated with a reduction in the occurrence of macroalbuminuria (hazard ratio: 0.77; 95% CI: 0.68–0.87; *p* < 0.0001). Additionally, no significant difference was observed between dulaglutide and placebos in the requirement for continuous renal replacement therapy [67]. In the SUSTAIN-6 study, semaglutide was also associated with reduced incidence rates of new or worsening nephropathy (hazard ratio: 0.64; 95% CI: 0.46–0.88; *p* = 0.005). Furthermore, semaglutide was associated with a significantly reduced occurrence of macroalbuminuria (hazard ratio: 0.54; 95% CI: 0.37–0.77; *p* = 0.001). Finally, no significant difference was observed between semaglutide and placebo regarding the requirement for continuous renal replacement therapy [53].

In conclusion, semaglutide, liraglutide, and duraglutide were associated with benefits to composite renal outcomes, particularly a decreased incidence of macroalbuminuria, but were not significantly associated with other renal benefits, such as reduced estimated glomerular filtration rate (eGFR) deterioration or a reduced requirement for renal replacement therapy.

## 8. Conclusions

The GLP-1 RAs reviewed in this meta-analysis were associated with safety, weight loss, glucose reduction, CV outcomes, HF, and renal outcomes. In descending order of benefit to glycemic control, semaglutide was statistically superior to liraglutide, dulaglutide, albiglutide, 2 mg of exenatide weekly, and 10 µg of exenatide twice daily. This same order of superiority was observed with respect to associated weight loss. Moreover, these GLP-1 RAs all improved CV outcomes overall but had different associations with the incidence of individual MACES. In particular, oral 14 mg daily semaglutide and 1.8 mg daily injected liraglutide were associated with a reduced risk of CV death, although this benefit was not observed for the other GLP-1 RAs. Semaglutide was associated with superior outcomes for HF and other CV outcomes in non-diabetic patients with obesity; by contrast, liraglutide was associated with worse HF outcomes in patients with diabetes with a reduced ejection fraction. In terms of benefits to kidney function, semaglutide, dulaglutide, and liraglutide were associated with superior composite renal outcomes, reducing the occurrence of new or persistent macroalbuminuria; however, these GLP-1 RAs were not associated with benefits to the occurrence of eGFR deterioration or the requirement for continuous renal replacement therapy. Finally, GLP-1 RAs may provide additional benefits to patients with obesity or diabetes.

## Figures and Tables

**Table 1:** Characteristics of GLP-1 RAs.

GLP-1 RAs	First Approved Date	Amino Acid Sequence	Elimination Half-Life	Administration Schedule	Phase III Clinical Trial Program	Reference
For subcutaneous injection						
Exenatide	2005 (USA); 2006 (Europe);	Exendin-4	3.3–4.0 h	Twice daily	AMIGO	[26]
Liraglutide	2009 (Europe); 2010 (USA);	Mammalian GLP-1	12.6–14.3 h	Once daily	LEAD	[27]
Once-weekly exenatide	2012	Exendin-4	3.3–4.0 h	Once weekly	DURATION	[34]
Lixisenatide	2013 (Europe); 2016 (USA);	Exendin-4	2.7–4.3 h	Once daily	GetGoal	[35]
Dulaglutide	2014	Mammalian GLP-1	4.7–5.5 days	Once weekly	AWARD	[31]
Albiglutide	2014 (Europe);	Mammalian GLP-1	5.7–6.8 days	Once weekly	HARMONY	[36]
Semaglutide (SQ)	2017 (USA); 2019 (Europe);	Mammalian GLP-1	5.7–6.7 days	Once weekly	SUSTAIN	[20]
For oral administration						
Semaglutide (long-acting)	2020	Mammalian GLP-1	5.7–6.7 days	Once daily	PIONEER	[33]

**Table 2:** Comparison of associated reductions in blood glucose level and weight for various GLP-1 RAs.

	Active Comparators	Change in HbA1c	Change in Weight	Reference
DURATION-1	Exenatide 10 µg BID	−1.5	−3.6	[26]
	Exenatide 2 mg QW	−1.9%	−3.7	
	*p* value	0.0023	0.89	
DURATION-5	Exenatide 10 µg BID	−0.9	−1.4	[37]
	Exenatide 2 mg QW	−1.6	−2.3	
	*p* value	<0.0001	<0.05	
DURATION-6	Exenatide 2 mg QW	−1.28	−2.68	[38]
	Liraglutide 1.8 mg QD	−1.48	−3.57	
	*p* value	0.02	0.0005	
LEAD-6	Exenatide 10 µg BID	−0.79	−2.87 kg	[39]
	Liraglutide 1.8 mg QD	−1.12%	−3.24 kg	
	*p* value	<0.0001	0.22	
HARMONY 7	Albiglutide 50 mg QW	−0.78	−0.64	[40]
	Liraglutide 1.8 mg QD	−0.99	−2.16	
	*p* value	0.0846	<0.0001	
AWARD-6	Dulaglutide 1.5 mg QW	−1.42	−2.90	[41]
	Liraglutide 1.8 mg QD	−1.36	−3.61	
	*p* value	<0.0001	0.011	
SUSTAIN-3	Semaglutide 1.0 mg QW	−1.5	−5.6	[20]
	Exenatide 2 mg QW	−0.9	−1.9	
	*p* value	<0.0001	<0.0001	
SUSTAIN-10	Semaglutide 1.0 mg QW	−1.7	−5.8	[42]
	Liraglutide 1.2 mg QD	−1.0	−1.9	
	*p* value	<0.0001	<0.0001	

**Table 3:** Comparison of GLP-1 RAs with respect to cardiovascular outcomes.

Agent	Study	Median Follow-up	Prior CVD%	Primary Composite CV Outcome HR	*p* Value	Cardiovascular Death HR	*p* Value	Fatal or Nonfatal Myocardial Infarction HR	*p* Value	Fatal or Nonfatal Stroke HR	*p* Value
Efpeglenatide	AMPLITUDE-O	1.81	89.6	0.73	0.007	0.72	0.07	0.75	0.09	0.74	0.19
Semaglutide(oral)	PIONEER-6	1.3	85	0.79	0.17	0.49	0.021	1.04	0.49	0.76	0.43
Semaglutide	SUSTAIN-6	2.1	59	0.74	0.02	0.98	0.92	0.81	0.26	0.65	0.066
Albiglutide	Harmony	1.6	100	0.78	0.0006	0.93	0.58	0.75	0.003	0.86	0.3
Dulaglutide	REWIND	5.4	32	0.88	0.026	0.91	0.21	0.96	0.63	0.76	0.01
Lixisenatide	ELIXA	2.1	100	1.02	0.81	0.98	0.85	1.03	0.71	1.12	0.54
Liraglutide	LEADER	3.8	81	0.87	0.01	0.78	0.007	0.86	0.046	0.86	0.16
Exenatide	EXSCEL	3.2	73	0.91	0.06	0.88	0.096	0.97	0.62	0.85	0.095

**Table 4:** Comparisons of GLP-1 RAs with respect to renal outcomes.

Agent	Semaglutide	Dulaglutide	Liraglutide
Study	SUSTAIN-6	REWIND	LEADER
Median follow-up	2.1	5.4	3.8
Composite renal outcome HR	0.64	0.85	0.78
*p* value	0.005	0.0004	0.003
New onset of macroalbuminuria HR	0.54	0.77	0.74
*p* value	0.001	<0.0001	0.004
Sustained doubling of serum creatinine HR	1.28	0.89	0.89
*p* value	0.48	0.07	0.43
Need for continuous renal replacement therapy HR	0.91	0.75	0.87
*p* value	0.8	0.4	0.4

## Data Availability

The data presented in this study are available upon reasonable request from the corresponding author. The data are not publicly available due to ethical regulations of the database.
